# MiR-890 inhibits proliferation and invasion and induces apoptosis in triple-negative breast cancer cells by targeting CD147

**DOI:** 10.1186/s12885-019-5796-9

**Published:** 2019-06-13

**Authors:** Cheng Wang, Cheng Xu, Ruijie Niu, Guangfu Hu, Zhangyuan Gu, Zhigang Zhuang

**Affiliations:** 1 Department of Breast surgery, Shanghai First Maternity and Infant Hospital, Tongji University School of Medicine, Shanghai, 200040 China; 2 Department of Breast Surgery, Central Hospital of Huangpu District, Shanghai, 200020 China; 3 Department of Breast Surgery, Yangpu Hospital, Tongji University School of Medicine, Shanghai, 200090 China

**Keywords:** miR-890, CD147, TNBC, Proliferation, Invasion

## Abstract

**Background:**

Triple-negative breast cancer (TNBC) is a type of breast cancer with a high degree of malignancy. Because of the remarkable biological characteristics of high invasion, metastasis and recurrence, TNBC is often accompanied by a poor prognosis. As a molecular characteristic of TNBC, high expression of CD147 has been confirmed by a large number of studies. However, the mechanism of CD147 expression regulation in TNBC remains elusive. In this study, we investigated the roles of miR-890 in inhibiting CD147.

**Methods:**

Quantitative Reverse Transcription-Polymerase Chain Reaction (qRT-PCR) was used to detect CD147 mRNA and miR-890 level, and western blotting was used to detect CD147 protein. Bioinformatics screening and 3′-Untranslated Region (3′-UTR) luciferase assays were used to analyze the microRNAs (miRNA) binding site. Cell proliferation, apoptosis and invasion were assessed by using CCK-8, flow cytometry and transwell assays.

**Results:**

The upregulation of miR-890 inhibited cell proliferation and invasion, induced apoptosis in MDA-MB-231 and HCC-70 TNBC cells by negatively regulating its target gene, CD147, and the upregulation of CD147 rescued the inhibitory effects of miR-890. miR-890 targeted CD147 by binding to its 3′-UTR. Further results showed that the upregulation of miR-890 also inhibited the expression of MMPs, the downstream genes of CD147, and promoted the cleavage of Caspase-3. The CD147 recovery experiment was further confirmed by the activity changes in the downstream MMPs of CD147. In addition, it was confirmed that the effect of CD147 in promoting TNBC cell proliferation and invasion, inhibiting apoptosis was related to the change in caspase-3 activity.

**Conclusion:**

The downregulation of miR-890 is the potential cause of high CD147 expression in TNBC, which can promote the malignant transformation of TNBC.

## Background

Due to its characteristics of strong invasiveness, local recurrence, high distant metastasis rate and poor prognosis, triple-negative breast cancer (TNBC) is a high-risk disease that has always been a hot spot in breast cancer research in recent years [1]. CD147, also known as extracellular matrix metalloproteinase inducer (EMMPRIN), is a highly glycosylated transmembrane glycoprotein that is widely expressed in hematopoietic and nonhematopoietic cell lines and belongs to the immunoglobulin superfamily [2]. CD147 is often expressed at low levels in normal tissues and benign tumors and is highly expressed in malignant tumors, acting as a promoter in tumor invasion and metastasis, the malignant transformation of tumors and angiogenesis [3]. Studies have shown that CD147 can induce its effector cells (fibroblasts around the tumor, etc.) to produce matrix metalloproteinases (MMPs) and significantly improve the content and activity of MMPs in tumor tissues. The excessive degradation of interstitial components and collagen in the vascular basement membrane and connective tissue barrier is closely related to the occurrence, metastasis and prognosis of tumors [4, 5].

CD147 is upregulated in the tumor cells of breast cancer tissues, while it is less expressed in the carcinoma stroma [6]. The expression level of CD147 is positively correlated with the progression, TNM stage, lymph node metastasis and overexpression of human epidermal growth factor receptor 2 (Her-2) and is negatively correlated with the expression of estrogen receptor (ER) and progesterone receptor (PR) [7]. In different types of invasive breast cancer, the expression of CD147 is also significantly different and associated with the clinical invasive ability, which has been considered a risk factor for breast cancer recurrence and metastasis [8]. Our previous studies showed that the expression rate of CD147 in the TNBC subtype was up to 85.70% [9]. Thus far, the mechanism of CD147 upregulation in TNBC remains elusive. Through comparative analysis between TNBC tissues and cell transcription and CD147 protein expression, we found that high CD147 expression in TNBC was caused by inactivation of the posttranscriptional regulatory mechanism. The expression of miRNAs is significantly different between normal and tumor tissues, suggesting that miRNAs may play a potential role in tumorigenesis. Many studies have shown that miRNAs are related to tumorigenesis by regulating mRNA expression. By means of bioinformatics analysis, we deduced that miR-890 might be an important factor regulating the expression of CD147 mRNA.

In this study, we confirmed that miR-890 was a negative regulator of CD147 and that the downregulation of miR-890 led to the upregulation of CD147 in TNBC.

## Methods

### Cell culture

Human TNBC cell lines MDA-MB-231(TCHu227) and HCC-70(TCHu148) and human normal mammary epithelial cells MCF-10A(SCSP-575) were all purchased from the Cell Bank of the Chinese Academy of Sciences (CBCAS, Shanghai, China) and maintained in RPMI-1640 medium (Thermofisher, CA, USA) supplemented with 10% fetal bovine serum (FBS, Thermofisher). 293TN cells (LV900A-1) used for lentivirus-producing was purchased from System Biosciences (CA, USA) and were maintained in Dulbecco’s Modified Eagle’s Medium (DMEM, Thermofisher). All cells were passaged by 0.25% trypsin digestion (Invitrogen) and incubated in an cell culture incubator (Forma 3110,Thermofisher) at 37 °C in 5% CO2.

### Tumor tissues

Under the approval of the medical ethics committee of the Central Hospital of Huangpu District and conforming to the principles outlined in the Declaration of Helsinki for the use of human tissue or subjects and after obtaining the written informed consent from the patient,tumor tissues were collected from 20 TNBC patients who underwent breast surgery at the Central Hospital of Huangpu District, immediately snap frozen in liquid nitrogen, and stored at − 80 °C until RNA extraction. The age of patients ranged from 31 to 76 years, with a median age of 54 years. No patient was treated with neoadjuvant therapy before surgery. Immunohistochemistry confirmed that ER, PR and HER-2 of tumor tissues were negative. All patients were a nonspecific type of carcinoma (simple invasive ductal carcinoma), and other types of TNBC, such as large sweat adenocarcinoma, adenoid cystic carcinoma and metaplasia carcinoma, were excluded.

### Vectors construction

The coding sequence (CDS) of human CD147 (NM_001728.3) was amplified by using the primers 5′-GGAATTCGCCACCATGGCGGCTGCGCTGTTCG-3(forward)’ and 5′-CGGGATCCTCAGGAAGAGTTCCTCTGG-3′(reverse) with human cDNA template prepared by reverse transcription of total RNA isolated from 293TN cells and digested with EcoRI and BamHI (Takara,Dalian,China). The digested product was recovered by gelatinization and cloned into the expressing vector pcDH1-GFP (System Biosciences), the recombinant vector was named pcDH1- CD147.

Human genomic DNA was extracted from 293TN cells by the QIAamp DNA Mini Kit (Qiagen, Germany) according to the instructions and used as the template to amplify the precursor of miR-890 with the primers 5′-GGAATTCTTGAAATCACCTTGAGAACAC-3′ (forward) and 5′-CGGGATCC AAGATTAAGTTCAGGGTTCAGG − 3′(reverse). The PCR product (317 bp) was digested with EcoR I and BamH I and ligated into pcDH1-GFP to obtain the recombinant vector pcDH1-miR-890.

Human CD147’s 3′- untranslated region (UTR,406 bp) was amplified with human cDNA with the primers 5′-GCTCTAGAGGCAGGTGGCCCGAGGACGC-3′(forward) and 5′-GCTCTAGAAATGGCGCAACCAGACAGCATTGG-3′(reverse). The product was digested with Xba I (Takara) and inserted into the pGL3-promotor vector (Promega, MI, USA) to obtain recombinant plasmid pGL3-wt-CD147 which carry a wild type seed region located downstream of the luciferase gene. The seed region was mutated from 5′-TCCAAGT-3′ to 5′- AGTACTC-3 by point mutation and obtain the pGL3-mt-CD147 carry the mutant seed region, respectively. All insertion fragments of recombinant vectors were confirmed by DNA sequencing and the transformed strains were amplified and the endotoxin-free plasmid DNA were extracted by the EndoFree Plasmid Maxi Kit (Qiagen) according to the instructions.

### Luciferase assay

Bioinformatics online software TargetScan (http://www.targetscan.org, Whitehead Institute, Cambridge, MI, USA) was used to predict whether a seed region of miR-890 exists within the 3′-UTR of human CD147. The miR-890-mimics (5′- UACUUGGAAAGGCAUCAGUUGtt-3′), the miR-890-inhibitor (5′- CAACUGAUGCCUUUCCAAGUAtt-3′) and the negative control (NC, 5′- AGACGUGUAUCGUAACUGAUGtt − 3′) were obtained from Shanghai Sangon (Shanghai, China). A suspension of 293TN cells in logarithmic phase was prepared and seeded into 6-well plates at a concentration of 2 × 10^5^ cells per well and maintained in DMEM medium supplemented with 10% FBS at 37 °C in 5% CO2 for 24 h. The amount of DNA and RNA and the experimental procedure are strictly in accordance with the kit instructions of lipofectmaine2000 transfection reagent. 50 ng pGL-TK per well was used for the internal reference of luciferase relative analysis. 48 h after transfection, luciferase activity was measured by using the Dual-Luciferase® Reporter Assay System (Promega).

### Assessment and correlation analysis of miR-890 and CD147 in TNBC specimens and cell lines

20 pairs of TNBC tumors and paracarcinoma tissues, as well as MDA-MB-231, HCC-70 and MCF-10A cells (1× 10^6^ each), were collected and subjected to total RNA extraction and qRT-PCR for the measurement the levels of miR-890 and CD147 mRNA. Total protein extraction and western blotting were performed for the CD147 protein expression.

### Recombinant lentivirus preparation

293TN cells in logarithmic phase were seeded in 10 cm culture dishes at 1 × 105 cells by using DMEM medium containing 10% FBS and cultured at 37 °C in 5% CO2 for 24 h. 2 μg of recombinants vector and 10 μg of pPACK Packaging Plasmid Mix (System Biosciences) were co-transfected into 293TN using Lipofectamine2000 transfection reagent (Invitrogen) according to the manufacturer’s protocol. The supernatant was harvested and cleared by centrifugation at 5000×g at 4 °C for 5 min and then passed through a disposable filter (0.45 μm, Millipore, MI, USA) after 48 h of transfection. The virus titer was determined by a gradient dilution method. The recombinant lentivirus were named Lv-miR-890 and Lv-CD147 and stored at − 80 °C freezer after dispensing.

### Effect of miR-890 overexpression on the CD147 and MMP-9 and cleaved caspase-3 protein in TNBC cell lines

MDA-MB-231 and HCC-70 cells in logarithmic phase were seeded into 6-well plates at a density of 5 × 10^5^ cells/well in RPMI-1640 medium containing 10% FBS and cultured at 37 °C in 5% CO2. One day later, lentivirus (Lv-miR-890 or Lv-CD147) was added at an multiplicity of infection of 10. The infection efficiency was evaluated by observing the expression green fluorescent protein as the fluorescence marker 72 h after infection. Total RNA were isolated and subjected to qRT-PCR to examine the levels of miR-890, and protein were isolated and subjected to western blotting to examine the expression of CD147 and MMP-9 and cleaved caspase-3 protein, respectively.

### Cellular proliferation and apoptosis and invasion assay

MDA-MB-231 and HCC-70 infected with Lv-NC, Lv-miR-890 or Lv-miR-890 and Lv-CD147 for 72 h were trypsinized and seeded into 96-well plates at a density of 1 × 10^4^ cells per well. The cells were cultured at 37 °C in 5% CO2. Cell viability was examined using CCK-8 (Cell Counting Kit-8, Dojindo, Japan) at 24, 48, and 72-h timepoints according to the kit instructions. Apoptosis was assessed using flow cytometry (FACS Calibur, BD, USA) with the Annexin V: FITC Apoptosis Detection Kit II (Cat:556570, BD) according to the instructions. Cell invasion experiments were performed using a QCM™ 24-well Fluorimetric Cell Invasion Assay Kit (Chemicon International, MI, USA) according to the manufacturer′s instructions. 500 μl medium supplemented with 10% FBS was used as a chemoattractant. 72 h after virus infection, 1 × 10^5^ cells of each group were seeded into the upper chamber and cultured in medium supplemented with 1% FBS for 12 h under 37 °C and 5% CO2. After 12 h, cells that invaded the underside of the membrane were fixed in 4% paraformaldehyde and stained with crystal violet staining solution, and the number of cells was counted by a fluorescence assay according to the kit instructions. The grouping was the same as that in the proliferation assay.

### QRT-PCR

Total RNA was extracted from cells and tissues by trizol lysis method and quantificated by a UV spectrophotometry. 2 μg RNA of each sample was reverse transcribed to synthesize complementary DNA (cDNA) by using a M-MLV reverse transcription kit (Takara, Dalian, China). For miR-890 level assay, the specific primers: U6 snRNA (MI0005533) 5′-TACCTTGCGAAGTGCTTAAAC-3′ and miR-890(NR_004394.1) 5′-GTCGTATCCAGTGCGTGTCGTGGAGTCGGCAATTGCACTGGATACGATAAGAC-3′ were used, and the Random9 primer (Takara) were used for CD147 mRNA assay. PCR reactions were carried out by using SYBR Premix Ex Taq (TaKaRa, Dalian, China). The following primers were used for quantification: U6 snRNA-forward 5′-GTGCTCGCTTCGGCAGCACAT-3′ and U6 snRNA reverse 5′-TACCTTGCGAAGTGCTTAAAC-3′ with producing a segment of 112 bp; miR-890-forward 5′-GCCGGCGCCCGAGCTCTGGCTC-3′ and miR-890-reverse 5′-TACTTGGAAAGGCATCAGTTG-3’with producing a segment of 71 bp; CD147-forward 5′- TTGAAGGGCAGGGTCCCAA-3′ and CD147-reverse 5′-TGTCGATGGAGATGGTGCTGG-3′; β-actin-forward 5′-CCTGTACGCCAACACAGTGC-3′ and β-actin-reverse 5′- ATACTCCTGCTTGCTGATCC-3′. PCR systems were SYBR Premix Ex Tap 10 μl, pairs of primers (20uM) 0.2 μl each, and cDNA 2 μl, added with dH2O to 20 μl. Cycling parameters were as follows: 40 cycles of denaturation at 95 °C for 10 s, annealing at 60 °C for 20 s and extension at 72 °C for 20 s. Each RNA sample was run in triplicate. The relative levels of miR-890 and CD147 were normalized by using the 2ΔΔCt method, U6 snRNA and β-actin were used as the reference.

### Western blotting

Total protein was extracted from the cells and tumor tissues by using M-PER mammalian protein extraction reagent or T-PER tissue protein extraction reagent (Pierce, IL, USA) and estimated by a bicinchoninic acid protein assay kit (Pierce). Equal amounts of 20 μg protein were loaded onto (11%) SDS-PAGE gels and transferred onto nitrocellulose membranes. The blots were probed with a monoclonal antibody against human CD147 (1:500), MMP-9 (1:500), Caspase-3 (1:400), Caspase-3 cleaved (1:300) and β-actin (1:1000) (Abcam, Cambridge, UK), followed by the secondary HRP-conjugated anti-rabbit antibody (Abcam). After washing with Tris Buffered saline Tween (TBST,10X), the bands were detected by chemiluminescence (ECL) and imaged with X-ray films. β-actin was used as an endogenous reference for normalization.

### Statistical analysis

Data were analyzed by Student’s t-test using Windows XP Excel 2010 (Microsoft: Redmond, WA, USA, 2010). Probabilities (*p* value) < 0.05 were regarded statistically significant. All statistical analyses were performed using SPSS 18.0 software (IBM Corp., NY, USA).

## Results

### MiR-890 was downregulated and CD147 was upregulated in TNBC tumors and cell lines

**Fig. 1:**
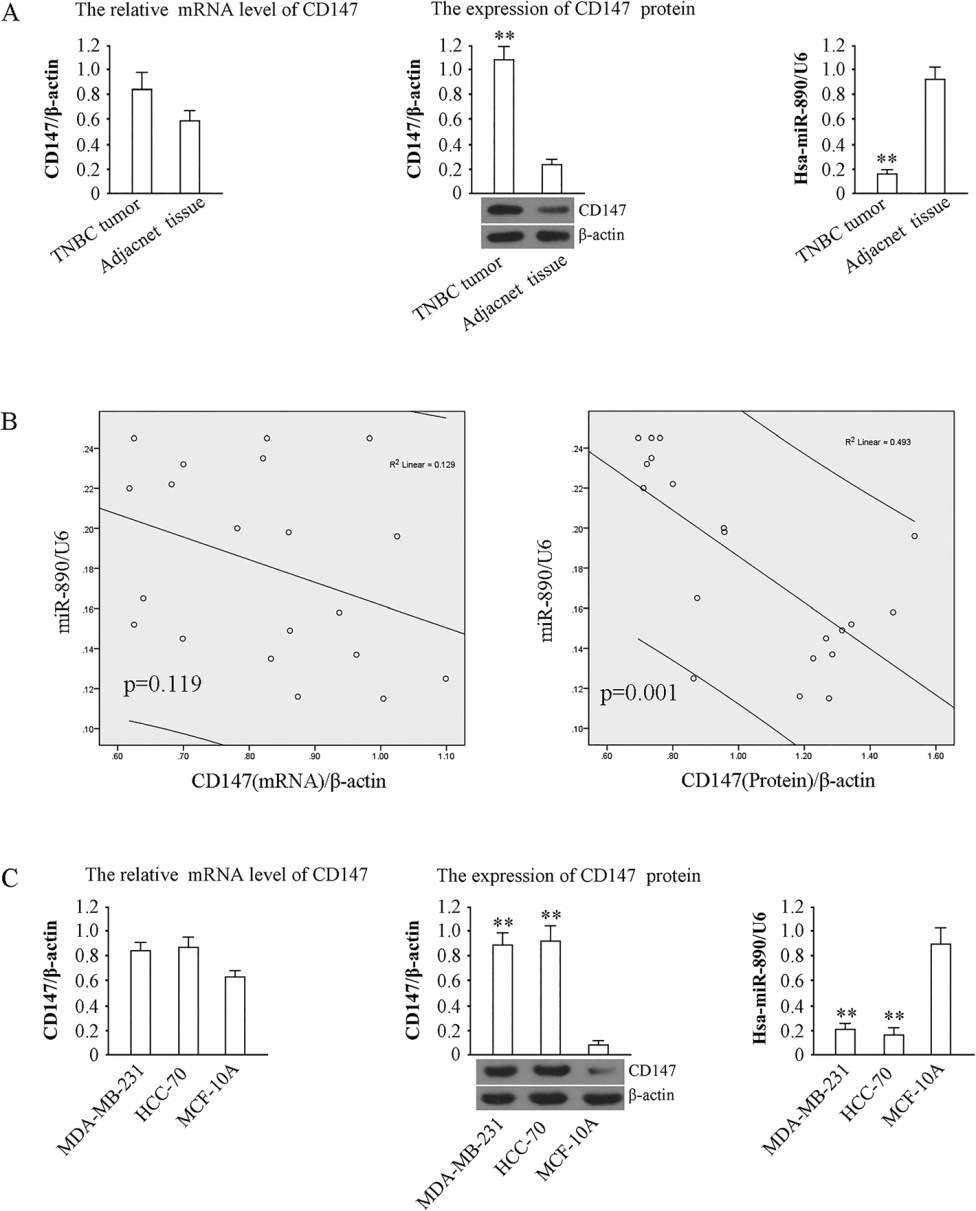
Levels of miR-890, CD147 mRNA and protein in TNBC tissue and cells. **a** Determination of CD147 mRNA, CD147 protein and miR-890 expression in 20 pairs of TNBC tissues and adjacent tissues. U6 and β-actin served as internal reference for the determination of miR-890 and CD147 mRNA, and the relative expression values of miR-890 and CD147 mRNA content in TNBC tissue was used. For the determination of CD147 protein expression, 20 pairs of samples were pooled, and tested by western blotting, β-actin served as internal reference. **b** Correlation analysis of CD147 protein/mRNA and miR-890 in 20 TNBC tumors. **c**. Determination of CD147(left), CD147 protein (middle,42 kDa) and miR-890 (right) in MDA-MB-231 and HCC-70 and MCF-10A cells. ** *P* < 0.01, vs. MCF-10A. The tests were carried out on three biological triplicates, and data are expressed as the mean ± SD

The results of quantitative PCR showed that the expression of miR-890 decreased in TNBC compared to adjacent tissues (*P* < 0.01), and western blot analysis showed that CD147 protein was higher in TNBC tumors than in adjacent tissues (*P* < 0.01). The levels of CD147 mRNA were slightly higher in TNBC tumors than in adjacent tissues, but there was no significant difference between the groups (*P* > 0.01) (Fig. 1a). Pearson Correlation analysis of CD147 protein or mRNA and miR-890 in TNBC was performed, and the data showed that in 20 TNBC tumor samples, miR-890 level was inversely correlated with CD147 protein (Correction coefficient = − 0.702, *P* = 0.001) but not CD147 mRNA (Correction coefficient = − 0.360, *P* = 0.119) (Fig. 1b). CD147 protein was also elevated in MDA-MB-231 and HCC-70 cells compared with that in MCF-10A cells (*P* < 0.01) (Fig. 1c), while miR-890 was weakly expressed in the TNBC cell lines. Together, these results suggest that miR-890 expression is negatively correlated with CD147 protein.

### MiR-890 inhibits CD147 expression by interacting with the 3′-UTR of CD147 mRNA

**Fig. 2:**
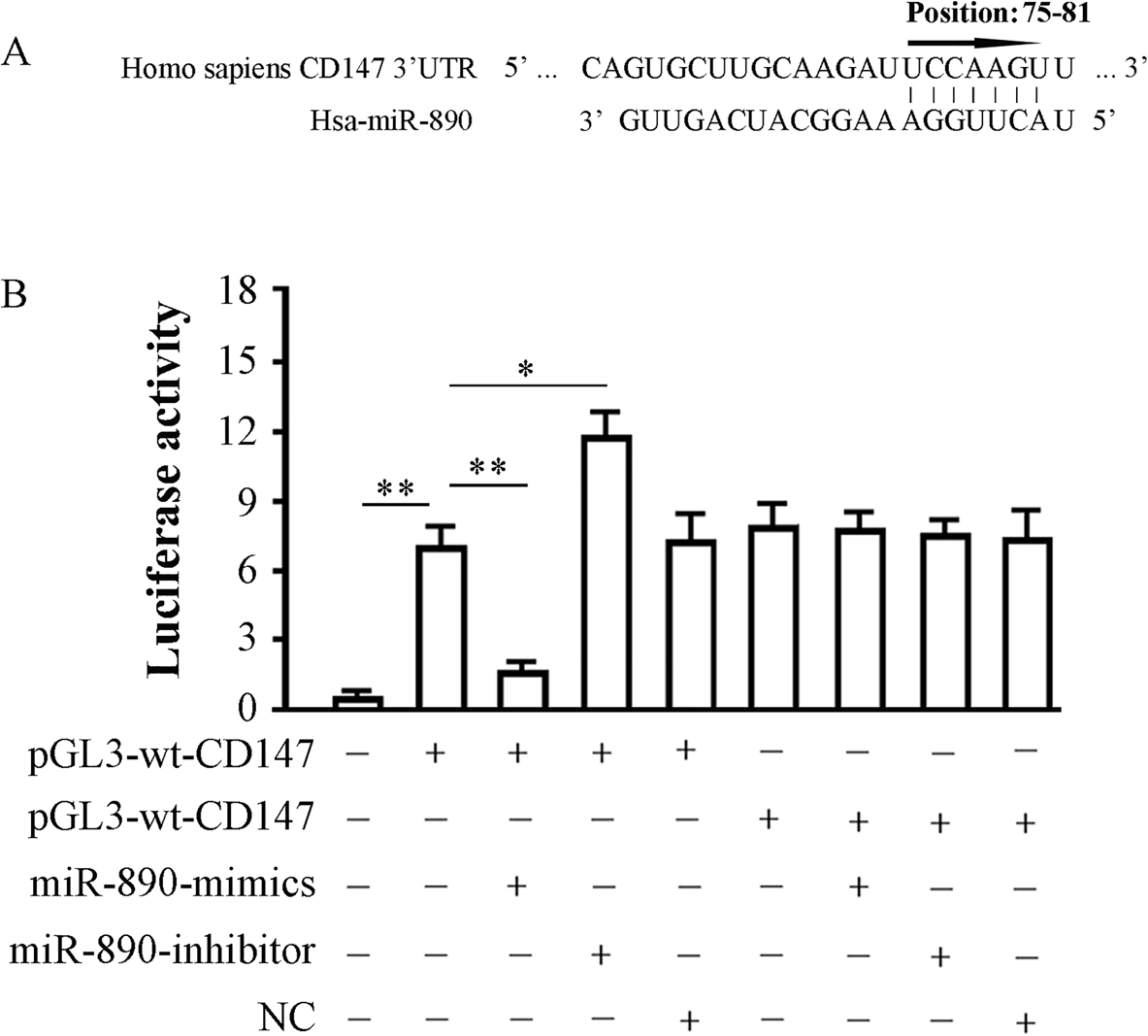
miR-890 binds to CD147 3’UTR. **a** Predicted binding site of miR-890 in 3′-UTR of CD147 gene; **b** Effects of miR-890 on the expression of a luciferase cassette with the CD147 3′-UTR. 293TN cells were transfected with pGL3-wt-CD147 or pGL3-mt- CD147 in the presence or absence of miR-890-mimic or inhibitor and subjected to luciferase activity assay 48 h later. The histogram shows the relative firefly luciferase activity for the different experimental groups. *, *P* < 0.05, and **, *P* < 0.01. Data are expressed as mean ± SD of at least three independent experiments

Bioinformatics analysis identified a seven-base miR-890 seed sequence in the 3′-UTR of CD147 mRNA (Fig. 2a). We therefore constructed luciferase reporter vectors of the 3′-UTR of CD147 mRNA in 293TN cells to verify whether this site represents a valid miR-890 target. Reporter vectors that contained the wild-type CD147 3′-UTR or a variant in which the miR-890 target site within the 3′-UTR had been mutated were generated. Both reporter constructs expressed luciferase at a high level. However, the miR-890 mimic significantly inhibited luciferase activity in cells transfected with the reporter vector that contained the wild type 3′-UTR (6.92 ± 0.81 vs. 1.93 ± 0.53; *P* < 0.01), while the miR-890 inhibitor significantly increased luciferase activity in these cells (6.92 ± 0.81 vs. 11.78 ± 0.96; *P* < 0.05). Conversely, in cells transfected with the reporter vector encoding the mutated miR-890 target site, neither the miR-890 mimic nor the miR-890 inhibitor had any observable effect on luciferase activity (*P* > 0.05). Cotransfection with miR-890-NC (nontargeting control) had no effect on the luciferase activity of either of the vectors (*P* > 0.05). These results verified the presence of a miR-890 target site in the 3′-UTR of CD147 mRNA and demonstrated that the binding of miR-890 to this target site inhibited CD147 expression.

### Effect of miR-890 and CD147 expression via a lentiviral approach in TNBC cells

**Fig. 3:**
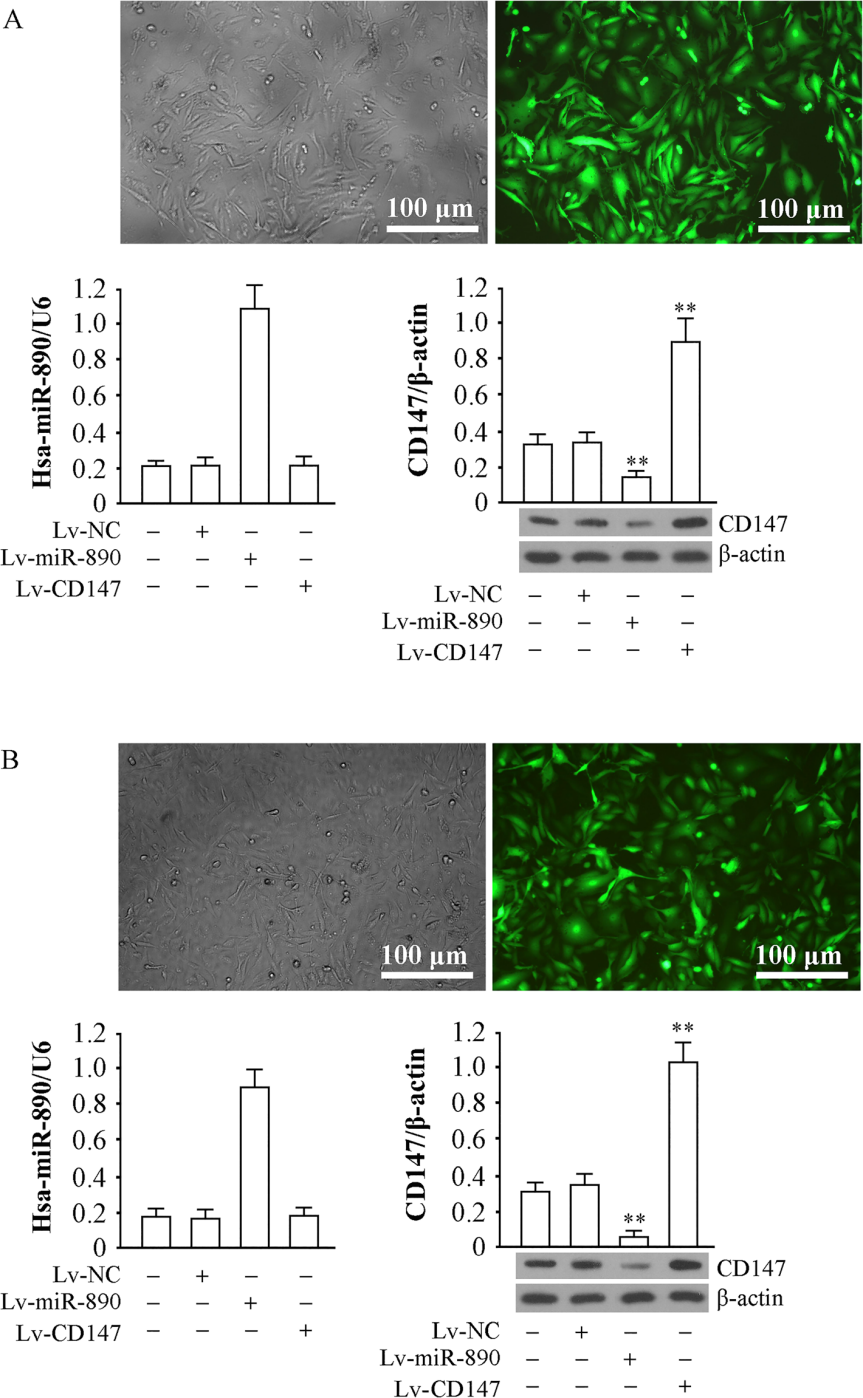
Genetic intervention through a lentiviral approach and interaction detection. **a** GFP expression 72 h after MDA-MB-231 was infected with recombinant virus. The infection rate was estimated by observing the number of the cells expressing GFP in each view. Determination of miR-890 by qRT-PCR with U6 served as internal reference, and the CD147 protein level was detected by western blotting with β-actin (37 kDa) served as an internal reference (down). **b** The same experimental data appeared in HCC-70. ** *P* < 0.01. The tests were carried out on three biological triplicates, and data are expressed as the mean ± SD

The recombinant lentiviruses Lv-NC, Lv-miR-890 and Lv-CD147 were used to infect MDA-MB-231 and HCC-70 cells. GFP (green fluorescent protein) was detected in most of the cells 72 h after infection, which suggested that the gene delivery efficiency was sufficient in the two TNBC cell lines (Fig. 3a). miR-890 was significantly increased by Lv-miR-890 (*P* < 0.01) and did not change in cells infected with Lv-CD147 (*P* > 0.05); CD147 protein levels were significantly increased by Lv-CD147 and decreased by Lv-miR-890 (*P* < 0.01) (Fig. 3b). These findings suggest that miR-890 upregulation can downregulate CD147 protein expression in MDA-MB-231 and HCC-70 cells and that the overexpression of CD147 has no obvious effect on miR-890.

### The overexpression of miR-890 inhibits cellular proliferation and invasion and induces apoptosis in TNBC cell lines

**Fig. 4:**
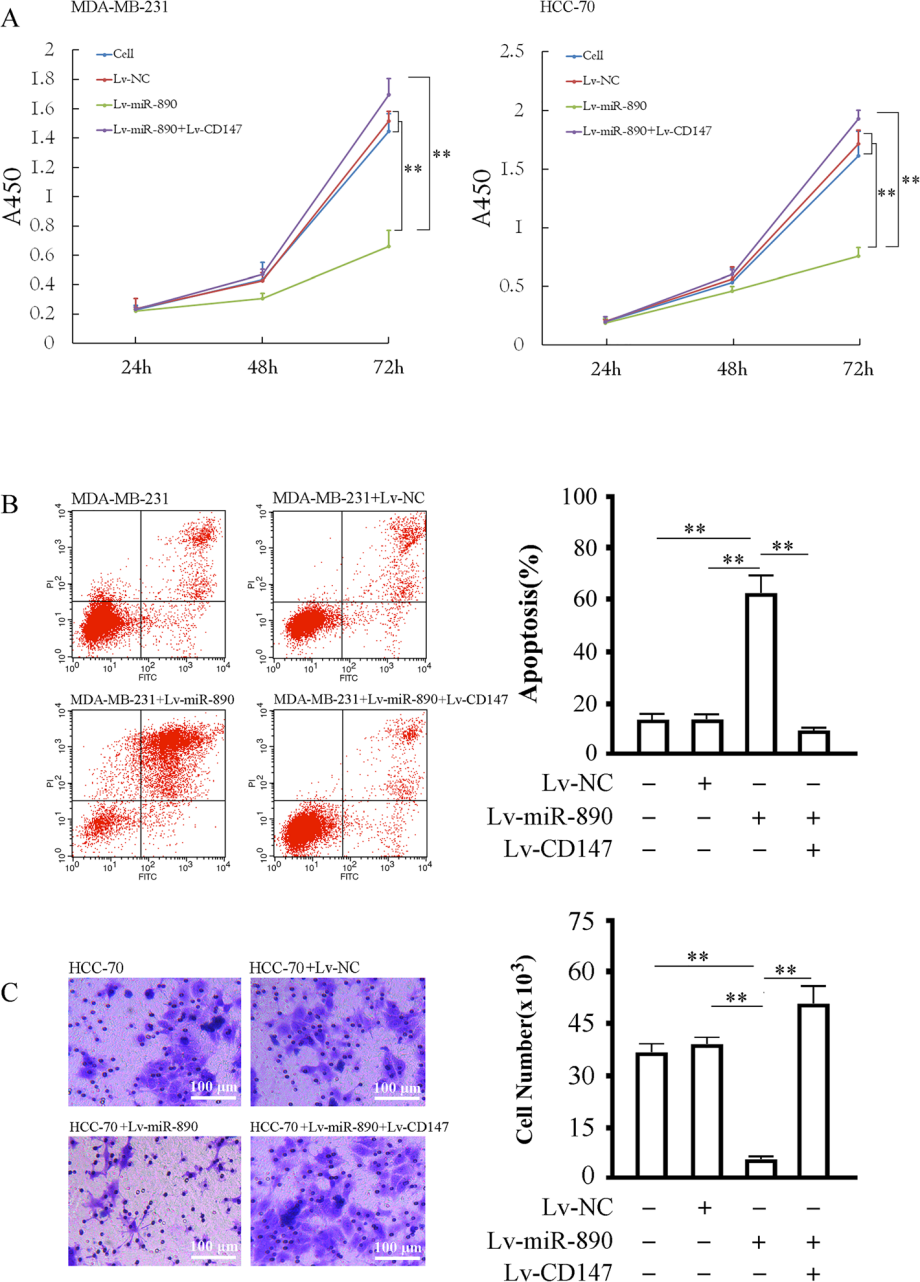
Effects of miR-890 upregulation on the proliferation, apoptosis and invasion in TNBC cells. **a** The proliferation of MDA-MB-231 and HCC-70 cells 24–72 h after being infected with the indicated virus determined by CCK-8 assay. **b** Apoptosis in MDA-MB-231 infected with indicated lentivirus. Left panel: representative plots of MDA-MB-231 undergoing indicated treatments, the x-coordinate is the FITC channel and the y-coordinate shows PI staining, the lower left panel represents living cells, the lower right panel shows early apoptotic cells, the upper left panel shows necrotic cells and the upper right panel shows late apoptotic cells. Right panel: Quantification of apoptosis for the indicated treatments (including early and late apoptotic). **c** Invasion data of the HCC-70 cells 72 h after being infected with the indicated virus determined by a transwell assay.** *P* < 0.01, *P < 0.05. The tests were carried out on three biological triplicates, and data are expressed as the mean ± SD

MDA-MB-231 and HCC-70 cells infected with Lv-miR-890 or Lv-CD147 were subjected to a proliferation assay. The results suggested that the upregulation of miR-890 inhibited the proliferation of MDA-MB-231 and HCC-70 cells (*P* < 0.05). Moreover, Lv-CD147 infection significantly weakened the inhibitory effect of miR-890 on the proliferation of the two cell lines. Cells infected with Lv-NC or with Lv-miR-890 and Lv-CD147 did not show remarkable morphological changes, and there was no difference between the three groups (*P* > 0.05) (Fig. 4a). The apoptosis assay in MDA-MB-231 cells revealed that the apoptosis rates in the four cell groups (untreated cells, Lv-NC, Lv-miR-890 and Lv-miR-890 combined with Lv-CD147) were 17.01 ± 2.11%, 16.94 ± 2.03%, 59.12 ± 7.63% and 7.51 ± 1.01%, respectively, with a significant difference between the cell group and the Lv-miR-890 group (*P* < 0.01), the Lv-NC group and the Lv-miR-890 group (*P* < 0.01), and the Lv-miR-890 combined with the Lv-CD147 group and the Lv-miR-890 group (*P* < 0.01), but no difference between the cell group and the Lv-NC group (*P* > 0.05) or between the Lv-NC group and the Lv-miR-890 combined with Lv-CD147 group was observed (*P* > 0.05)(Fig. 4b). Furthermore, cell invasion assay results demonstrated that miR-890 expression inhibited invasion in HCC-70 cells (*P* < 0.01), and CD147 overexpression could reverse the invasion inhibition caused by miR-890 expression (*P* < 0.05) (Fig. 4c).

### Effect of miR-890 on the expression of MMP-9 and cleavedcaspase-3

**Fig. 5:**
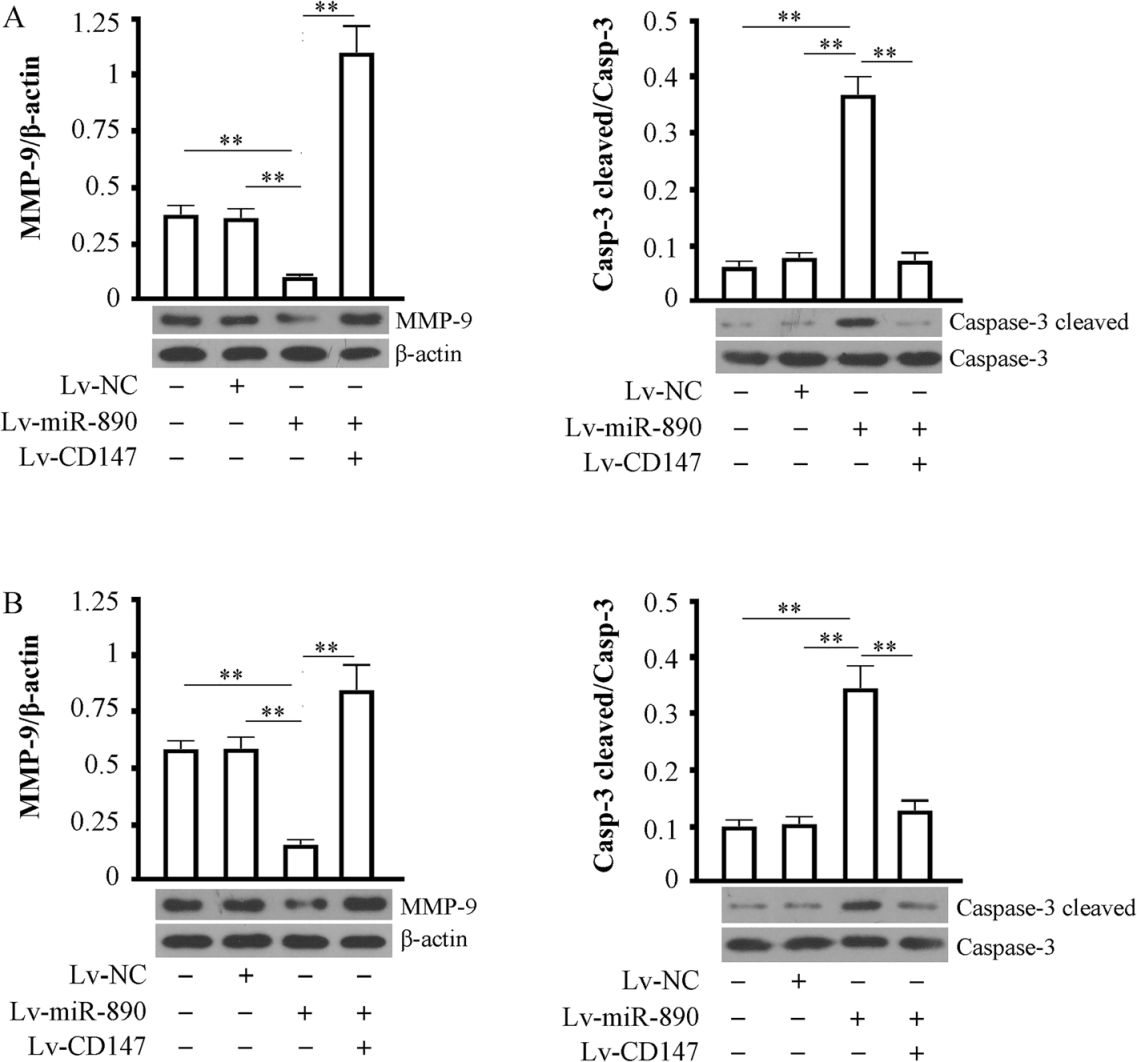
Effects of miR-890 expression on MMP-9 and Caspase-3 cleaved. **a** MDA-MB-231 and HCC-70 infected with or without Lv-miR890 or Lv-CD147,72 h post infection were used for detecting MMP-9 (78 kDa) and Caspase-3 cleaved (17 kDa). β-actin as a loading control. Data were representative of at least three independent experiments. ** *P* < 0.01. **b** The same experimental data appeared in HCC-70

We assessed MMP-9 expression and cleaved caspase-3 levels in MDA-MB-231 (Fig. 5a) and HCC-70 (Fig. 5b) cells overexpressing miR-890 combined with cells overexpressing CD147 or not. The results showed that MMP-9 was significantly increased and cleaved caspase-3 was significantly decreased by miR-890 expression (*P* < 0.01 vs cell control or NC control groups). However, the changes in the two proteins were both blocked by the overexpression of exogenous CD147 (*P* < 0.01 vs cells infected with Lv-miR-890).

## Discussion

Breast cancer is one of the most common malignant tumors in women worldwide, accounting for approximately 9% of malignant tumors. According to the expert conference of St. Gallen in 2013, breast cancer can be divided into four types according to different immunohistochemical characteristics: luminal A, luminal B, overexpression of Her-2 and TNBC [1]. As a type of high-risk breast cancer with a special molecular subtype, TNBC accounts for 10~17% of all breast cancer, with unique clinical features and biological behaviors, including strong invasiveness, high local recurrence and distant metastasis rate and poor prognosis. Due to the lack of effective therapeutic targets, effective adjuvant therapy for TNBC is very scarce except for chemotherapy. Researchers are conducting various studies to identify new key genes regulating the malignant biological behavior of TNBC, to clarify the pathogenesis of specific protein molecules in the pathogenesis of TNBC, and to identify molecular targets that can be applied for clinical treatment [10–12].

CD147 is highly expressed in hematopoietic and nonhematopoietic cell transmembrane glycoproteins and belongs to the immunoglobulin superfamily. CD147 is a matrix metalloproteinase inducer on the surface of tumor cells that induces the release of matrix metalloproteinases, degrades matrix metalloproteinases, and promotes the infiltration and metastasis of tumor cells [2]. CD147 is widely distributed in the human body; however, its expression is low in normal tissues and in benign pathological tissues [13]. Only in some precancerous lesion tissues and cancer tissues is its expression level significantly increased (overexpressed). High expression of the CD147 gene has been found in some malignant cells, such as lung cancer, ovarian cancer, liver cancer, brain glioma, oral squamous epithelial cancer and osteosarcoma tissue [14–17]. Most studies have suggested that the combination of CD147 and its effector cells (fibroblasts around the tumor, etc.) can induce the production of MMPs and significantly improve the content and activity of MMPs in tumor tissues. The excessive degradation of interstitial components and collagen in the vascular basement membrane and connective tissue barrier is closely related to the occurrence of metastasis and prognosis of tumors, which is an important area of clinical research and for the accurate treatment of TNBC [18, 19].

Studies have suggested that CD147 is highly expressed in breast cancer tissues and is mainly expressed in the membranes and cytoplasm of tumor cells [20]. With the development of breast cancer, the expression of CD147 is continually increased, which corresponds to its clinical invasive ability. CD147 is regarded as a risk factor for the recurrence and metastasis of breast cancer. For patients with breast cancer, the expression of CD147 protein is significantly higher in those with distant metastasis than in those without metastasis. In cell lines, the expression of CD147 is higher in cells with metastatic ability than in inert cells [5, 21]. The survival time of patients with positive CD147 protein is significantly lower than those with negative expression [22–25]. The upregulation of CD147 in MDA-MB-435 breast cancer cells promoted tumor growth, infiltration and metastasis in nude mice, and the expression of MMP-2 and MMP-9 increased in tumor cells [26]. The knockdown of CD147 in MDA-MB-435 cells by transfecting a CD147 siRNA eukaryotic expression vector resulted in the downregulation of VEGF, hypoxia inducer 1α, and MMP-2, which significantly promoted cell apoptosis [27]. Previous studies have shown that the expression of CD147 in TNBC was closely associated with histologic grade, p53 and Ki-67 index. The positive expression of CD147 in TNBC was up to 81.82%, which indicated that CD147 was involved in the relevant molecular mechanism of highly invasive biological characteristics of TNBC and suggested that CD147 expression played important roles in the differentiation and proliferation of tumor cells [9]. Our previous research found that the expression level of CD147 protein was an independent prognostic factor of DFS and OS in TNBC patients [9]. In cell lines, the expression of CD147 is higher in breast cancer cells with metastatic ability [8]. Although the role of CD147 in promoting the progression of breast cancer has been widely investigated, the mechanism regulating CD147 expression in the special classification of TNBC remains elusive.

In our study, the quantitative detection of CD147 mRNA in TNBC cells and clinical specimens showed that the mRNA of the CD147 gene in TNBC tissues and in TNBC cells did not show a significant difference from that of adjacent tissues and normal human mammary epithelial cells. Western blotting results revealed that CD147 protein in TNBC tissues and TNBC cells was greatly elevated; therefore, it was anticipated that the expression of CD147 was regulated at posttranscriptional levels.

Posttranscriptional regulation is the regulation of gene expression after RNA transcription, which is one of the characteristics of eukaryotic gene expression [28, 29]. As one of the classical postreduction regulation mechanisms, the miRNA could combine with the 3′-UTR of the target gene to inhibit protein translation [30]. miRNAs are endogenous noncoding small molecule RNAs that play an important role in the pathophysiological process of organisms by regulating gene expression. miRNAs consist of 18–25 nucleic acids, which can pair with the base specific to the noncoding region of the target gene, leading to its degradation or translation repression, thus regulating the transcription of genes and playing an important role in regulating life activities. miRNAs have been reported to be involved in the formation and progression of TNBC by inhibiting their target genes [31, 32]. By means of bioinformatics analysis, we deduced that miR-890 might be an important factor regulating the expression of CD147 mRNA. At present, little is known about the function of miR-890. It has been reported to be involved in the regulation of the inflammatory response. miR-890 was also identified as a potential prognostic biomarker for pancreatic ductal adenocarcinoma and is involved in the development of prostate cancer [33, 34]. Our study revealed that miR-890 was downregulated in TNBC tissues and cell lines and that CD147 was the miR-890 target gene. Furthermore, miR-890 was verified to target the 3′-UTR of CD147. In vitro experiments, the overexpression of miR-890 through the lentivirus system could effectively inhibit the expression of CD147 and the proliferation of TNBC cell lines. The analysis of a set of CD147 gene regression experimental groups showed that the inhibitory effect of miR-890 on TNBC was achieved through the target gene CD147. Subsequent studies confirmed that miR-890 upregulation indirectly promotes caspase-3 and decreases MMP-9 by inhibiting CD147, which can further affect TNBC cell apoptosis and invasion.

On the basis of the previous functional research of CD147 in TNBC, we performed a preliminary analysis of the downstream route action and the upstream factors of CD147. Our research revealed that miR-890 is an endogenous inhibitor of CD147 in TNBC and is a potential tumor suppressor. CD147 could promote the progression of TNBC randomly occurring through cell cycle regulation, which indicates that miR-890 is a valuable target for the clinical treatment of TNBC. Of course, the sudden decrease in miR-890 content in TNBC could be from an emergency or another cause, which requires more in-depth study.

## Conclusion

The downregulation of miR-890 in TNBC leads to the upregulation of CD147 and promotes the proliferation and invasion, inhibits apoptosis of TNBC cells. The overexpression of miR-890 can effectively inhibit the proliferation and invasion, induce the apoptosis of TNBC cells. This study is a profound systematic attempt to analyze the cause of high CD147 expression in TNBC, while miR-890 was proven as a potential target for TNBC gene therapy.

## Data Availability

The datasets used and/or analysed during the current study available from the corresponding author on reasonable request.

## Abbreviations

3′-UTR: 3′-Untranslated Region
ER: Estrogen receptor
Her-2: Human epidermal growth factor receptor-2
MMPs: Matrix metalloproteinases
PR: Progesterone receptor
qRT-PCR: Quantitative Reverse Transcription-Polymerase Chain Reaction
TNBC: Triple-negative breast cancer
